# Diagnostic Algorithm to Subclassify Atypical Spitzoid Tumors in Low and High Risk According to Their Methylation Status

**DOI:** 10.3390/ijms25010318

**Published:** 2023-12-25

**Authors:** Jose Francisco González-Muñoz, Beatriz Sánchez-Sendra, Carlos Monteagudo

**Affiliations:** 1 Skin Cancer Research Group, Biomedical Research Institute INCLIVA, 46010 Valencia, Spainsansenbe@uv.es (B.S.-S.); 2 Department of Pathology, University of Valencia, 46010 Valencia, Spain

**Keywords:** Spitzoid melanocytic tumors, Spitz, melanoma, methylation, algorithm, novel biomarkers

## Abstract

Current diagnostic algorithms are insufficient for the optimal clinical and therapeutic management of cutaneous spitzoid tumors, particularly atypical spitzoid tumors (AST). Therefore, it is crucial to identify new markers that allow for reliable and reproducible diagnostic assessment and can also be used as a predictive tool to anticipate the individual malignant potential of each patient, leading to tailored individual therapy. Using Reduced Representation Bisulfite Sequencing (RRBS), we studied genome–wide methylation profiles of a series of Spitz nevi (SN), spitzoid melanoma (SM), and AST. We established a diagnostic algorithm based on the methylation status of seven cg sites located in *TETK4P2* (Tektin 4 Pseudogene 2), *MYO1D* (Myosin ID), and *PMF1-BGLAP* (PMF1-BGLAP Readthrough), which allows the distinction between SN and SM but is also capable of subclassifying AST according to their similarity to the methylation levels of Spitz nevi or spitzoid melanoma. Thus, our epigenetic algorithm can predict the risk level of AST and predict its potential clinical outcomes.

## 1. Introduction

Cutaneous spitzoid tumors constitute a heterogeneous subgroup of melanocytic neoplasms with variable malignant potential, ranging from benign Spitz nevus (SN) to spitzoid melanoma (SM), with potential metastatic dissemination [1]. However, there is a challenging intermediate diagnostic group called Spitz melanocytomas/atypical Spitzoid Tumors (AST) [2]. SN are melanocytic neoplasms composed of large epithelioid and/or spindled melanocytes with a regular architecture, usually symmetrical, sharply circumscribed, and typically less than 6 mm in diameter [2]. SM is a melanoma with an initiating genomic alteration characteristic of SN, larger than 6 mm and often greater than 10 mm, more deeply invasive, and showing greater cellular atypia with asymmetry, color variegation, or ulceration [2]. AST is a melanocytic neoplasms characterized by one or more atypical features (at least 5 mm and often greater than 10 mm in diameter, may be asymmetric, with irregular borders, and not well circumscribed) and is genetically intermediate between SN and SM [2].

Current diagnostic algorithms are insufficient for the proper diagnosis and prognosis of AST, which affects the optimal clinical and therapeutic management of these patients. In fact, clinicians often overdiagnose malignancy, resulting in unnecessary use of resources and treatment, with severe emotional and physical impacts on the patients and their relatives [3]. Therefore, predicting the metastatic potential of AST and the clinical outcome of these patients is a major challenge for clinicians [4].

Various approaches have been used over the years to correctly categorize and prognosticate ASTs, including immunohistochemical techniques (*p16*, *ki67* or *HMB45*) [4]; analysis of copy number variations (CNV) by comparative genomic hybridization (CGH) [5]; fluorescence in situ hybridization (FISH) [6,7]; genetic point mutations [8] in genes, such as *HRAS* [9,10], *NRAS* [9], *BRAF* [11] or *TERT* [12,13]; and kinase fusions [14] in genes such as *ROS1* [15], *ALK* [16] and *NTRK1* [17]. In fact, a diagnostic algorithm for ASTs based on the combination of immunohistochemical techniques, FISH and CGH has been described [18], although the diagnostic value of FISH for AST tumors is limited due to their heterogeneity; and lower than for SN and SM [4,6,19].

However, these molecular techniques are not capable of predicting the clinical behavior of AST and are insufficient for optimal clinical and therapeutic management. It has been proposed that epigenetic changes, which are heritable and reversible events that modify gene expression without changing the DNA sequence, can be used as new biomarkers [6,8,20]. DNA methylation is an epigenetic event that involves the addition of a methyl group to the fifth carbon of a cytosine nucleotide. It is typically associated with stable transcriptional silencing and plays an important role in several biological processes associated with development and disease, such as cell differentiation and regulation of gene expression [21]. In fact, in conventional melanoma, DNA methylation changes have been described to serve as diagnostic, prognostic, and therapeutic biomarkers, and it has been noted that DNA methylation is involved in tumor progression and prognosis [22,23,24]. These DNA methylation changes can be used as epigenetic biomarkers to diagnose and classify spitzoid tumors.

Although the methylation profiles of genes commonly affected in melanoma have been studied using the multiple ligand–dependent probe amplification (MLPA) technique, these limited methylation studies have not been able to identify relevant genes for AST classification [25,26]. As MLPA only allows the methylation study of single genes, it is necessary to assess the global genome methylation status of cutaneous spitzoid melanocytic tumors. Recently, global methylation profiles have been described to discriminate benign spitzoid tumors from conventional nevi and melanomas [27]. Here, using a methylation array, we found that genome–wide methylation levels of SN were similar to those of benign conventional nevi, but the Leukocyte UnMethylation for Purity (LUMP) score of SN was comparable to that of conventional melanoma. The LUMP score was used to estimate the leukocyte content in tumor samples and is a surrogate marker of leukocyte/lymphocyte infiltration [28]. In addition, SN showed homogeneous methylation in comparison to conventional melanoma, which presented a heterogeneous profile that was suggested to be related to its malignant behaviour [27]. However, the latter study only compared SN with conventional melanomas and did not include other spitzoid lesions such as AST and SM. We aimed to diagnose and classify all spitzoid tumors correctly.

Therefore, we used reduced representation bisulfite sequencing (RRBS) to determine the genome–wide methylation profile of the full spectrum of spitzoid lesions. Chatterjee et al. previously used RRBS to identify DNA methylation changes that could serve as progression markers [23] or their relationship with *PD-L1* expression [29] but only used primary and metastatic melanoma cell lines. In this way, we found differences between them and constructed a molecular algorithm to classify AST according to their similarity to benign or malignant spitzoid lesions.

## 2. Results

### 2.1. Differential Methylation Analysis

**Figure 1:**
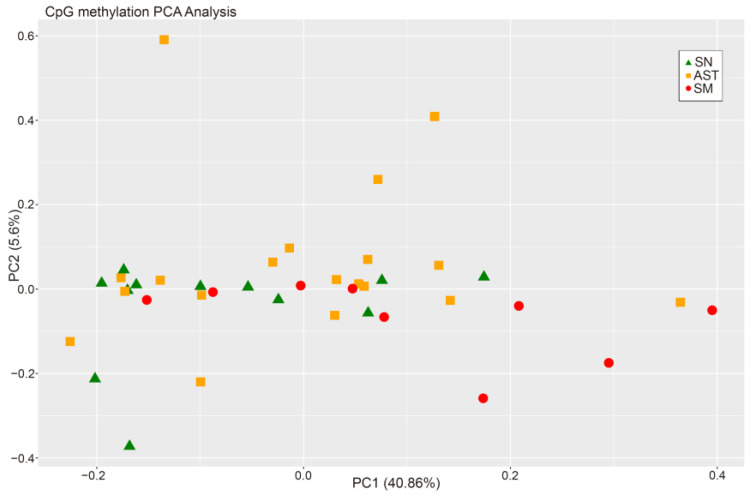
PCA from CpG methylation analysis of the three experimental groups.

When comparing the methylation status of SN, AST, and SM samples at the same time, there were no differences between the groups (Figure 1).

**Figure 2:**
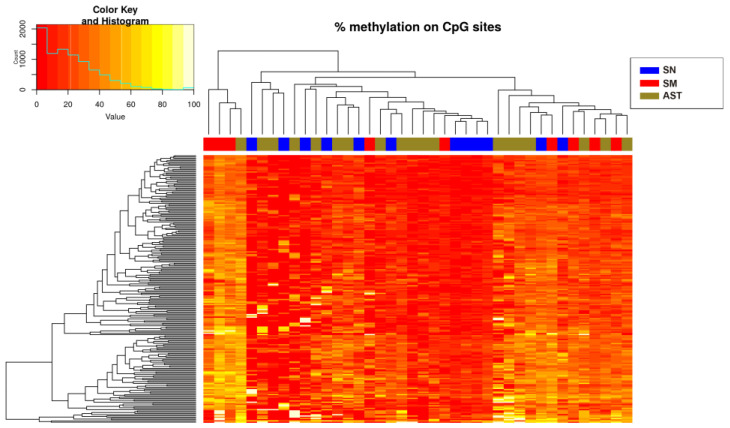
Heatmap showing the sites (214) with more than 10% methylation difference with respect to the control (SN) (*q*-value < 0.05 when comparing the three experimental groups). There is a histogram in the color key showing the number of cg sites (count) and the methylation percentage (value).

**Figure 3:**
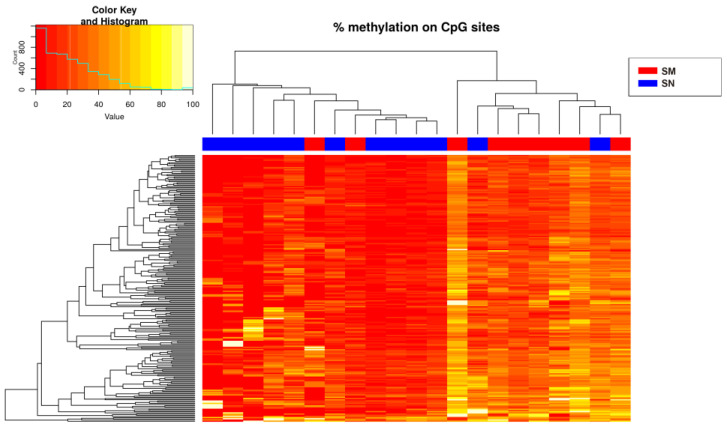
Heatmap showing the sites (214) with more than 10% methylation difference with respect to the control (SN) (*q*-value < 0.05 when comparing the Spitzoid nevi and melanoma groups). There is a histogram in the color key showing the number of cg sites (count) and the methylation percentage (value).

Only hypermethylated sites (214 sites) were detected in some chromosomes with a significance of *q*-value < 0.05 and a methylation difference higher than 10%. The heatmap of the significant sites showed that there was no separation between SN and SM (Figure 2) because ASTs included samples of unknown malignant potential. By studying only the methylation status of SN and SM tumors, differences between them were defined (Figure 3), and 224 sites were detected, including hypomethylation and hypermethylation.

### 2.2. Predictive Equations

To predict the clinical behavior of the AST group, the results obtained from the differential methylation analysis between the SN and SM were used to build the prediction algorithms.

**Figure 4:**
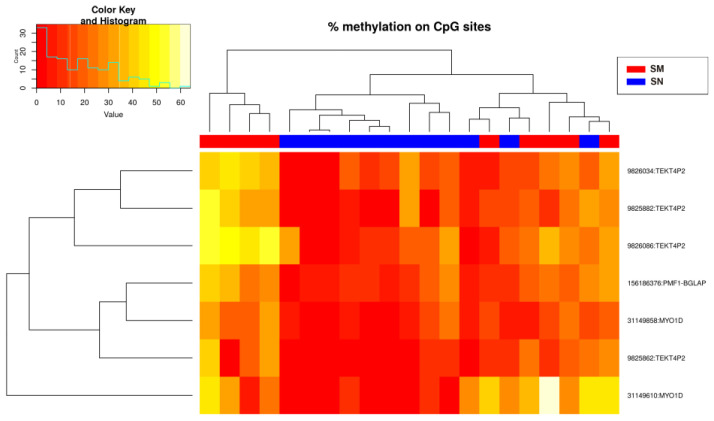
Heatmap showing the methylation levels of the seven methylation sites with *p*-value < 0.02 (Spitzoid nevi and melanoma groups). There is a histogram in the color key showing the number of cg sites (count) and the methylation percentage (value).

Modeling using binary logistic regression was performed for each of the 224 CpG sites. This analysis resulted in the selection of seven CpGs (*p*-value < 0.02) for the construction of the predictive algorithms: cg9825862, cg9825882, cg9826034, cg9826086, cg31149610, cg31149858, and cg156186376 (Figure 4), located in the genes *MYO1D* (Myosin ID), *TEKT4P2* (Tektin 4 Pseudogene 2), and *PMF1-BGLAP* (PMF1-BGLAP Readthrough) (Table 1).

**Figure 5:**
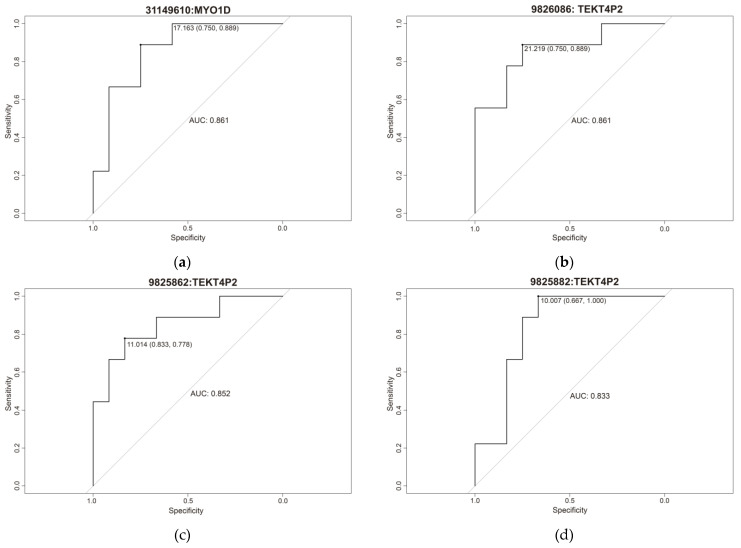

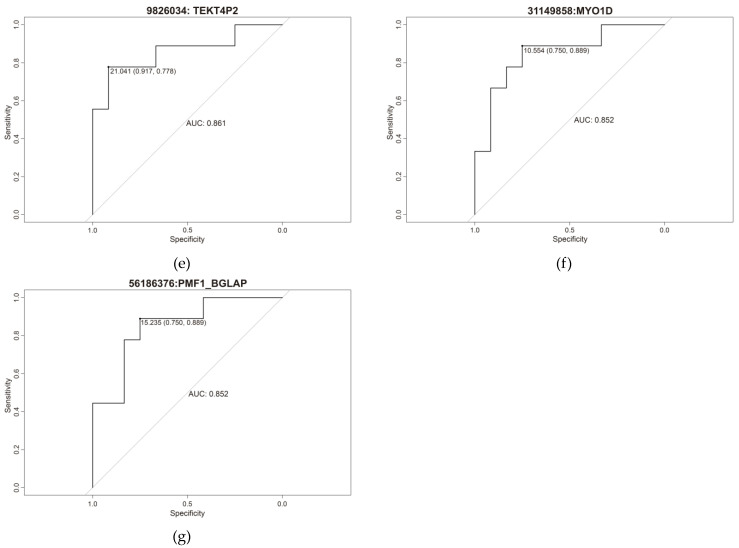
Individual ROC curve of (**a**) cg31149610, (**b**) cg9826086, (**c**) cg9825862, (**d**) cg9825882, (**e**) cg9826034, (**f**) cg31149858 and (**g**) cg156186376.

Receiver operating characteristic (ROC) curve analysis was performed to determine the predictive ability of each of the seven methylation sites (Figure 5), yielding an area under the curve (AUC) rank of 0.833–0.861 (minimum specificity of 0.667 and a minimum sensitivity of 0.833) (Table 2).

**Figure 6:**
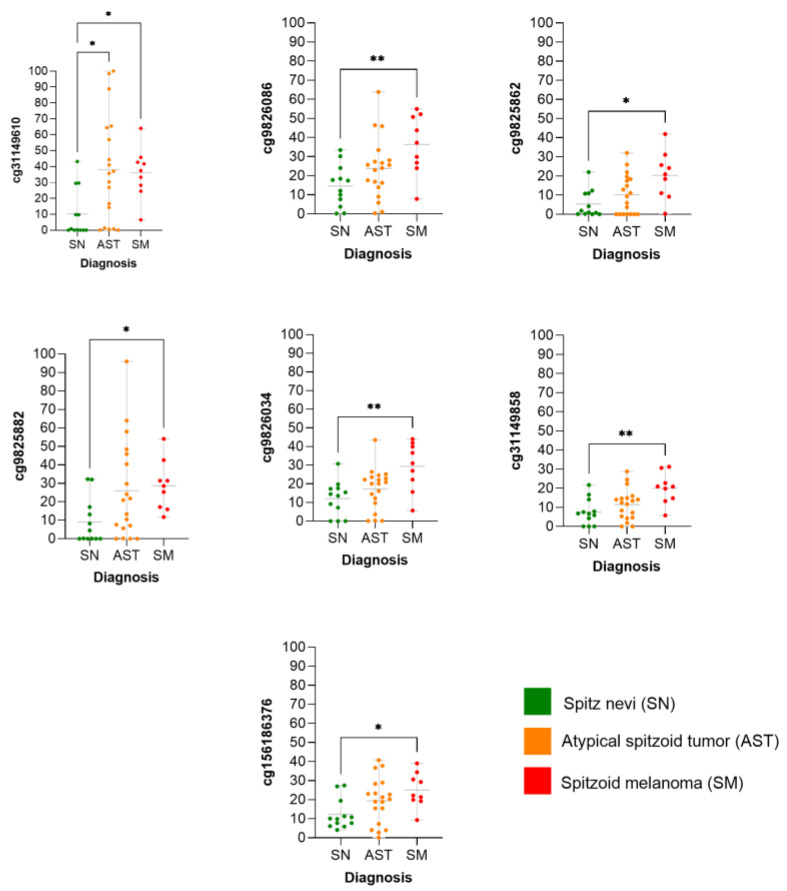
Methylation levels of individual CpG according to diagnosis. * = (*p*-value < 0.05); ** = (*p*-value < 0.01).

We observed that the methylation levels of the seven CpGs were significantly higher in SM than in SN (Figure 6), whereas the methylation status of the ASTs was scattered.

The first predictive algorithm was built with an equation that calculates the probability of being classified as melanoma Equation (1), where the indices from A to G are the percentages of methylation found in each of the seven sites included in the equation (A: cg9825862, B: cg9825882, C: cg9826034, D: cg9826086, E: cg31149610, F: cg31149858, G: cg156186376).

(1) $$P\left(Melanoma\right)=\frac{1}{\left(1+{exp}^{-\left(-342.3+3.4A-10.3B+3.4C+8.9D+8.0E+11.0F-8.7G\right)}\right)}$$

**Figure 7:**
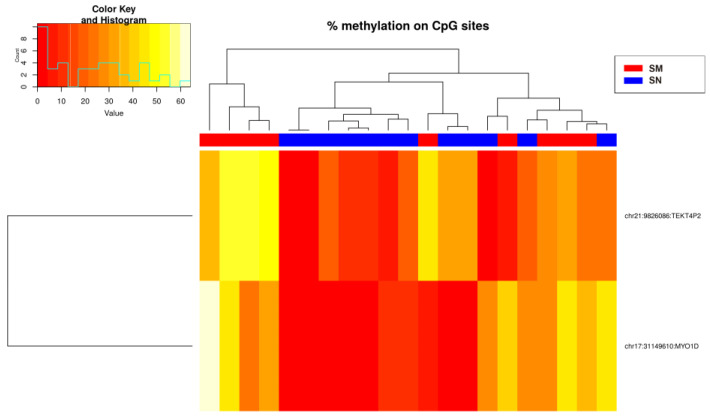
Heatmap showing the methylation levels of the two methylation sites from Equation (2) (Spitzoid nevi and melanoma groups). There is a histogram in the color key showing the number of cg sites (count) and the methylation percentage (value).

A second prediction algorithm Equation (2) was constructed using the glmulti R package version 1.0.7 to obtain the best predictive combination of the seven CpGs, resulting in cg31149610 (index E) and cg9826086 (index D) (Figure 7):

(2) $$P\left(Melanoma\right)=\frac{1}{\left(1+{exp}^{-\left(-0.16-0.012E+0.014D\right)}\right)}$$

### 2.3. Risk Prediction of the Samples

For both algorithms, we obtained a probability value named P(melanoma). If P(melanoma) < 0.4, the spitzoid tumor is classified as benign (SN) and therefore has no risk of clinical aggressiveness. Conversely, if P(melanoma) is ≥0.4, the spitzoid tumor is classified as having a high risk of clinical aggressiveness (SM).

Hence, a conservative false-positive characteristic is assumed because the model would rather err by saying that a spitzoid lesion would behave as an SM when it is not than classify a true SM as an SN.

Regarding the correct classification of the samples used for the construction of the predictive algorithms, Equation (1) was capable of classifying 100% of the samples in the correct group (SN and SM) with a *p*-value of 0.0002. In other words, the identified epigenetic signature makes it possible to classify melanoma cases with 100% sensitivity and Spitz nevi cases with 100% specificity. A total of 52.63% of AST cases were classified as SN (10/19), whereas 47.37% were classified as SM (9/19).

**Figure 8:**
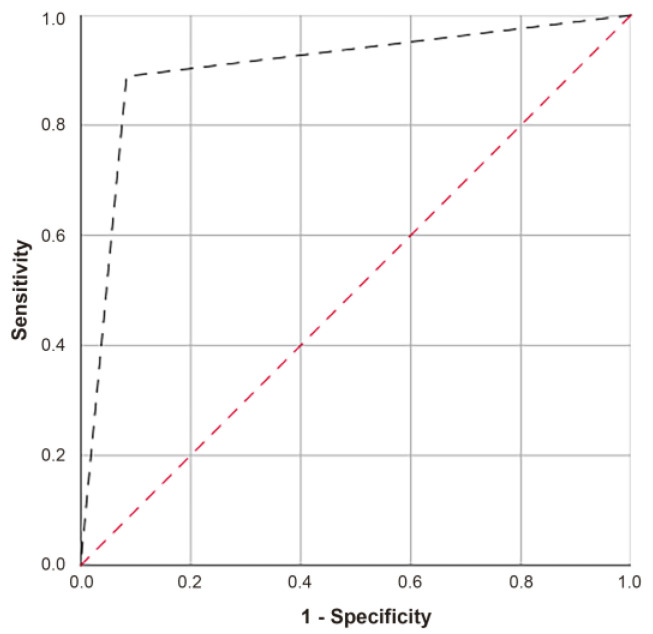
ROC curve of Equation (2).

A ROC curve was obtained to evaluate the ability of the second algorithm to correctly classify spitzoid tumors, showing an AUC of 0.903 (IC 95% 0.75–1.00) with a *p*-value of 0.002, sensitivity of 88.9%, and a specificity of 91.7% (Figure 8). Equation (2) failed to classify one SN and SM (Table 3). Here, 36.84% of the ASTs were classified as SN (7/19) and 63.16% as SM (12/19).

Although both algorithms coincided in the prediction of 84.21% of our samples, three AST values predicted as SN in Equation (1) were classified as SM in Equation (2) (Table 4).

## 3. Discussion

Although several biomarkers, techniques, and molecular algorithms (based on immunohistochemistry, FISH, and CGH [18]) have been proposed in recent decades to correctly classify, diagnose, and predict the clinical behavior of spitzoid tumors, there is still no widely accepted and effective clinical procedure to accomplish this task, largely due to the heterogeneity of AST tumors [4,6].

As previously described [27], SN lesions showed a different methylation profile compared to benign and malignant conventional melanocytic tumors, but the differences within the whole spitzoid tumor spectrum have not been investigated. Global methylation patterns and LUMP scores have been used to distinguish SN from conventional melanocytic tumors, but these tumors already have clear histological differences and are routinely correctly diagnosed by pathologists. In our study, we focused on the differences between SN, AST, and SM.

RBBS was performed because it can be applied to FFPE samples with small amounts of DNA and has a large genomic coverage. In addition, RBBS does not have batch effects compared with microarrays. Whole genome bisulfite sequencing (WGBS) was discarded because of its high sequencing costs and DNA requirements.

Here, we demonstrated a differential methylation status between SN and SM by observing 224 cg sites that were differentially methylated using RRBS. We found seven CpGs with a methylation difference higher than 10% and a *q*-value < 0.02 between Spitzoid samples located in *MYO1D* (2 sites), *TEKT4P2* (4 sites) and *PMF1-BGLAP* (1 site) genes.

Using this information, we created two predictive bioinformatics models based on methylation status to first distinguish SN and SM and additionally to subclassify AST according to their potential risk of clinical aggressiveness as a function of the probability value we called P(melanoma). Low risk means that the tumor has a low probability of behaving like an SM, i.e., SN lesion behavior is predicted when P(Melanoma) is less than 0.4. Conversely, if P(melanoma) is greater than or equal to 0.4, the lesion has a high risk of behaving like an SM lesion. In this way, a conservative false–positive character is adopted; that is, the model prefers to err by saying that a spitzoid lesion would behave as SM and not really before it would be a real SM and classify it as an SN.

Although the sensitivity and specificity of predictive Equation (2) (88.9% and 91.7%, respectively) were lower than those of Equation (1) (sensitivity and specificity of 100%), this improved the classification of each of the seven sites individually. Although the AUC of Equation (2) was 0.903, the AUC values of the individual sites ranged from 0.833 to 0.861.

Considering the classification power of Equation (1) for the known samples, we conclude that the two CpG sites from Equation (2) explain most of the variance in the predictive model, and the five additional CpG sites from Equation (1) improve the classification.

The *TEKT4P2*, *MYO1D*, and *PMF1-BGLAP* genes, which are involved in Equation (1), have been described in several tumors, but there is no information related to spitzoid lesions.

*TEKT4P2* is a pseudogen that has been described as a progression biomarker in cutaneous conventional melanoma [30]. It has been seen that *TEKT4P2* levels decrease as the stages increase (I/II and III/IV compared with stage 0), and additionally, *TEKT4P2* interacts with several miRNAs associated with the development of cutaneous melanoma, such as miR-193-3p, miR-194-3p or miR-194-5p [30]. Therefore, similar to conventional cutaneous melanoma, methylation of the non–coding RNA *TEKT4P2* could cause gene silencing and regulate other epigenetic markers, such as miRNAs in SM.

*MYO1D* encodes an unconventional myosin protein involved in actin filament organization. High levels of *MYO1D* have been described as a poor prognostic biomarker in urothelial cancer but as a good prognostic biomarker in renal cancer [31]. Thus, *MYO1D* may have dual activity. In fact, although a low expression of *MYO1D* is related to progression in prostate cancer due to hyperactivation of histone *H3K27me3* [32], an overexpression of *MYO1D* levels in colorectal cancer and acute myeloid leukemia causes an increase in *EGFR* expression, activating oncogenic pathways, and therefore promoting tumor progression [33,34]. Although it has been described that *H3K27me* expression is associated with melanocytic lesions, there is no information regarding its role in spitzoid tumors and its relationship with *MYO1D* methylation [8,35].

The locus *PMF1-BGLAP* is the read-through transcription site between *PMF1* and *BGLAP*. Its expression has been described as an unfavorable prognostic marker in liver cancer and renal cancer [31]. *PMF1-BGLAP* encodes *PMF1* (polyamine-modulated factor 1), a protein regulated by polyamines involved in chromosome alignment and segregation during mitosis [36]. The methylation of *PMF1* in bladder cancer patients has been associated with poor clinical outcomes [37]. Because *PMF1-BGLAP* is important during mitosis, increased *PMF1-BGLAP* methylation in SM may be associated with a higher mitotic ratio [2].

Previous studies using RRBS or WGBS in melanoma cell lines described that methylation differences between primary and metastatic melanomas at *TBC1D16* and *EBF3* were associated with tumor progression [23,38,39]. However, among the 224 cg differentially methylated sites, when comparing spitzoid tumors, we did not find differences in *TBC1D16* and *EBF3* methylation levels.

Thus, the predictive algorithms disclosed herein have high predictive power and offer a solution in the field of personalized precision medicine [40] to the current clinical challenge posed by patients with spitzoid melanocytic tumors currently classified as “of uncertain malignant potential,” thus providing a precise, clear and personalized answer to the diagnosis and prognosis of these patients [4,8,41]. In fact, despite the cost of RRBS and the difficulty of using this technology in health centers, using this algorithm in clinical practice may help pathologists properly diagnose spitzoid tumors regardless of their experience with these lesions. This epigenetic algorithm could avoid the use of invasive, expensive, and/or time-consuming techniques for both diagnosis and therapy (such as sentinel lymph node studies, PET, or adjuvant therapies) for lesions with a low risk of clinical aggressiveness while minimizing psychosocial and emotional distress to patients [42,43,44].

Nevertheless, it is recommended that a molecular approach that is more accessible to the current healthcare system than RRBS is found to determine the methylation status of the seven cg sites. Due to the complexity of the regions where these cg are located, cytosine and guanine are enriched; thus, at this moment, it is not possible to design primers for direct bisulfite pyrosequencing, methylation-specific PCR (MS-PCR) or MassARRAY (combination of competitive PCR with MALDI-TOF). It is possible that the technical and economic requirements for RRBS or WGBS will soon become lower and more affordable in routine clinical practice. Other approaches could be used, such as anchor-based bisulfite sequencing (ABBS) [45] and nanopore sequencing [46].

In conclusion, our newly identified epigenetic biomarkers and associated algorithms, whether or not combined with immunohistochemistry [18], FISH [7], NGS [47], or even other epigenetic markers [48,49], will improve the diagnosis and prediction of clinical outcomes in challenging ambiguous spitzoid tumors [50].

## 4. Materials and Methods

### 4.1. Human Samples

For this study, 40 formalin–fixed paraffin–embedded (FFPE) tumor samples from patients with spitzoid lesions were selected and classified according to the 5th Edition of the World Health Organization (WHO) Classification of Skin Tumor [2] as 12 SN, 19 AST, and 9 SM. Tumor specimens were collected at the time of surgery and reported to the Department of Anatomic Pathology of the Hospital Clínico Universitario, Valencia (Spain) from 1990 to 2018. The Ethical and Scientific Committees of the Hospital Clínico Universitario approved this protocol. Written informed consent was obtained from all the patients. The essential clinicopathological features are shown in Table 5.

### 4.2. Nucleic Acid Extraction

The pathologist selected the most representative tumor areas of each lesion from hematoxylin–eosin stained slides. These areas were manually punched from paraffin blocks to achieve at least 90% tumor cellularity.

Genomic DNA and total RNA (including small RNAs) from the same punch were simultaneously extracted using AllPrep DNA/RNA FFPE (Qiagen Cat# 80234, Hilden, Germany), following the manufacturer’s recommendations. The main advantage of this kit is its ability to simultaneously obtain genomic DNA and RNA from the same tumor region. This allows different techniques to be performed on small and rare lesions such as spitzoid tumors.

Briefly, deparaffinization was performed efficiently using a commercial solution (Qiagen Cat# 19093, Hilden, Germany), avoiding further washing steps. After solubilization, both nucleic acids were treated separately to remove formaldehyde crosslinks and then purified automatically using the QIAcube nucleic acid purification system (Qiagen Cat# 9002864, Hilden, Germany). RNA and DNA were eluted using 30 µL nuclease–free water and low-EDTA buffer and stored at −80 and −20 °C, respectively.

Both DNA and RNA were quantified using a Nanodrop One (Thermo Fisher Scientific, Waltham, MA, USA). DNA was isolated for the RRBS study, and RNA was extracted for future studies. As required for RRBS, the DNA concentration of the samples was also measured using the Qubit dsDNA BR Assay Kit (Thermo Fisher Scientific, Waltham, MA, USA), and the quality was assessed using the Fragment Analyzer and the DNF-487 Standard Sensitivity or the DNF-488 High Sensitivity Genomic DNA Analysis Kit (Advanced Analytical, Ames, USA), according to the sample concentration.

### 4.3. Reduced Representation Bisulfite Sequencing (RRBS)

RRBS enables genome–wide DNA methylation mapping with good resolution and theoretical coverage of 3.5 and 4 million CpG dinucleotides in the human genome. This methodology covers CpG islands, promoter regions, and other functional elements, including enhancers, CpG island boundaries, and non–coding RNAs.

DNA methylation profiling (RRBS Service) (Diagenode Cat# G02020000, Seraign, Belgium) was performed. The service workflow includes DNA quality control (QC), preparation of RRBS libraries, deep sequencing, and primary bioinformatics analysis [51].

RRBS libraries were prepared using the Premium Reduced Representation Bisulfite Sequencing Kit, according to the manufacturer’s protocol (Diagenode Cat# C02030033, Seraign, Belgium) [52]. First, DNA was digested with the restriction enzyme MspI, which recognizes CCGG sites, resulting in genomic fragments that start and end with a CpG dinucleotide, regardless of DNA methylation status. For library preparation, the ends were prepared, adaptors were ligated, and samples were selected by size.

Following library preparation, samples were pooled together into groups of eight and treated with bisulfite. Bisulfite converts non–methylated cytosines into uracils, which are then read as timines. Bisulfite treatment was performed under minimum DNA degradation conditions and maximum conversion efficacy of non-methylated cytosines. Next, the library was enriched by amplification via PCR, and a clean-up was performed using 1.45x beads: sample ratio of Agencourt^®^ AMPure^®^ XP (Beckman Coulter, Pasadena, CA, USA).

The Qubit dsDNA HS Assay Kit (Thermo Fisher Scientific, Waltham, MA, USA) was used to measure the DNA pool concentration, and their profiles were checked using the High Sensitivity DNA chip for 2100 Bioanalyzer (Agilent). In case of too high adapter dimer peaks, the pools were size selected one more time using 1.45x beads: sample ratio of Agencourt^®^ AMPure^®^ XP (Beckman Coulter, Pasadena, CA, USA), and quality control steps were performed again. RRBS library pools were sequenced on a HiSeq3000 (Illumina, San Diego, CA, USA) using 50 bp single-read sequencing (SR50).

### 4.4. Bioinformatics Analysis

Quality control of the sequencing was performed using FastQC version 0.11.8 (Babraham Bioinformatics, Babraham Institute, Cambridge, UK). Trim Galore! Version 0.4.1 was used to remove the adapters (Babraham Bioinformatics, Babraham Institute, Cambridge, UK). Reads were then aligned to the human reference genome hg19 using Bismak v0.16.1, followed by methylation calling using the corresponding Bismark functionality [53]. These quality controls were performed using the Diagenode RRBS Service (Diagenode, Seraign, Belgium).

In addition, other quality controls were performed using FastQC and BAM files using three independent programs: Bismark v0.22.1 [53], MultiQC V1.7 [54], and Qualimap v2.2.1 [55]. Normalization was performed using the normalizeCoverage function of the methylKit R package version 1.20.0 [56].

Differential methylation analysis was performed using the methylKit R package, applying Fisher’s exact test to obtain the *p*–value and q–value for each cg site. Predictive models were executed by independent logistic regression with the glm function of the R Stats package and the glmulti (version 1.0.7) function to find the best combination of the seven methylation sites of Equation (1) to build Equation (2) [57].

### 4.5. Statistical Analysis

Statistical analyses were performed using GraphPad Prism V.6.01 (GraphPad Prism Software, Inc., San Diego, CA, USA) and R 4.2.1. The following R packages were used: Stats for heatmap graphs, ROC curves and Kruskal–Wallis rank. A *p*–value of <0.05 was considered statistically significant.

## 5. Conclusions

In conclusion, we propose an algorithm with high predictive power for spitzoid tumors according to the methylation status of the seven CpGs described, which may be a key factor to classifying spitzoid tumors and predicting the risk of AST in the challenging group in a reliable and reproducible manner, and consequently to anticipate their potential clinical outcome.

## 6. Patents

Results from this work have been patented with an European Patent Application (application number 21382569.8) and an International Patent Application (application number PCT/EP2022/067556) entitled “Molecular tools for the diagnosis and prognosis of melanocytic Spitzoid tumors”, whose inventors are the authors of this manuscript.

## Figures and Tables

**Table 1:** Differentially methylated CpGs were selected for the construction of the predictive algorithms.

Position (cg)	Chromosome	Gene	Transcript
31149610	17	*MYO1D*	NM_015194
31149858	17	*MYO1D*	NM_015194
9826086	21	*TEKT4P2*	NR_038327
9825862	21	*TEKT4P2*	NR_038327
9825882	21	*TEKT4P2*	NR_038327
9826034	21	*TEKT4P2*	NR_038327
156186376	1	*PMF1-BGLAP*	NM_001199662

**Table 2:** Specificity, sensitivity, and area under the curve (AUC) of the 7 cg differentially methylated.

Position (cg)	Gene	Specificity	Sensitivity	AUC	Cut-Off
31149610	*MYO1D*	0.75	0.889	0.861	17.163
31149858	*MYO1D*	0.75	0.889	0.852	10.554
9826086	*TEKT4P2*	0.75	0.889	0.861	21.219
9825862	*TEKT4P2*	0.833	0.778	0.852	11.014
9825882	*TEKT4P2*	0.667	1	0.833	10.007
9826034	*TEKT4P2*	0.917	0.778	0.861	21.041
156186376	*PMF1-BGLAP*	0.75	0.889	0.852	15.235

**Table 3:** Prediction of Equations (1) and (2) of SN and SM tumor samples.

Sample	Diagnosis	Prediction 1	Prediction 2
1	SM	SM	SM
2	SM	SM	SM
3	SM	SM	SN
4	SM	SM	SM
5	SM	SM	SM
6	SM	SM	SM
7	SM	SM	SM
8	SM	SM	SM
9	SN	SN	SN
10	SN	SN	SN
11	SN	SN	SN
12	SN	SN	SN
13	SN	SN	SN
14	SN	SN	SN
15	SN	SN	SN
16	SN	SN	SM
17	SN	SN	SN
18	SM	SM	SM
19	SN	SN	SN
20	SN	SN	SN
21	SN	SN	SN

**Table 4:** Prediction of Equations (1) and (2) of AST samples.

Sample	Diagnosis	Prediction 1	Prediction 2
22	AST	SN	SN
23	AST	SN	SN
24	AST	SN	SM
25	AST	SM	SM
26	AST	SN	SN
27	AST	SM	SM
28	AST	SN	SN
29	AST	SM	SM
30	AST	SM	SM
31	AST	SM	SM
32	AST	SN	SN
33	AST	SM	SM
34	AST	SM	SM
35	AST	SM	SM
36	AST	SM	SM
37	AST	SN	SN
38	AST	SN	SM
39	AST	SN	SM
40	AST	SN	SN

**Table 5:** Clinicopathologic characteristics of the three subgroups of spitzoid tumors.

Diagnosis	n	Age (Years) at Diagnosis (Median ± SD ^#^)	Gender	Location	Diameter (mm) (Median ± SD ^#^)	Mitosis/mm^2^ (Median ± SD ^#^)
SN	12	24.08 ± 18.33	Male: 25% Female: 75%	Lower limb: 4 Trunk: 2 Upper limb: 4 Head & neck: 1 Not specified: 1	6.14 ± 3.18	0.33 ± 0.65
AST	19	20.6 ± 14.84	Male: 37.5% Female: 62.5%	Lower limb: 2 Trunk: 3 Upper limb: 3 Head & neck: 2 Not specified: 9	5.22 ± 2.48	1.53 ± 1.07
SM	9	47.22 ± 20.31	Male: 66.67% Female: 33.33%	Lower limb: 1 Trunk: 1 Upper limb: 4 Head & neck: 0 Not specified: 3	5.16 ± 1.31	5 ± 2.83

SN, Spitz nevus; AST, Atypical Spitzoid Tumor; SM, Spitzoid melanoma. ^#^ SD, standard deviation.

## Data Availability

The data presented in this study are available from the corresponding author upon reasonable request.
